# Digital Knowledge Translation Tools for Disseminating Sexual and Reproductive Health Information to Adolescents: Protocol for an Evidence Gap Map Review

**DOI:** 10.2196/55081

**Published:** 2024-02-13

**Authors:** Salima Meherali, Soumyadeep Bhaumik, Sobia Idrees, Megan Kennedy, Zohra S Lassi

**Affiliations:** 1 Faculty of Nursing College of Health Sciences University of Alberta Edmonton, AB Canada; 2 Meta-research and Evidence Synthesis Unit Health Systems Science George Institute for Global Health Sydney Australia; 3 Health Science Library University of Alberta Edmonton, AB Canada; 4 Robinson Research Institute Helen Mayo North Adelaide Australia

**Keywords:** adolescents, sexual and reproductive health, digital tools, knowledge translation, mobile phone

## Abstract

**Background:**

Digital or eHealth knowledge translation (KT) interventions have been identified as useful public health tools, particularly to advance sexual and reproductive health (SRH) among adolescents. Existing literature reviews on digital health interventions for adolescents’ SRH demonstrate limitations, including shortcomings in reporting and comprehensiveness that limit the utility and trustworthiness of findings. However, there is a lack of evidence synthesis on the effectiveness of available digital or mobile health KT tools to promote SRH interventions for adolescents.

**Objective:**

We aim to identify, map, and describe existing empirical evidence on the digital KT tools developed to improve adolescent SRH outcomes globally.

**Methods:**

This study will be conducted using an evidence gap map (EGM) approach to address the objectives, including reviewing relevant literature and a landscape analysis of the outcomes of interest. The following electronic databases will be searched for retrieval of literature: MEDLINE (1946-present), Embase (1974-present), and Global Health (1910-present) via OVID; CINAHL (1936-present) via EBSCOhost; Scopus (1976-present); and Cochrane Library (1993-present) via Wiley. We will include only those studies that focused on adolescents aged 10-19 years and addressed SRH outcomes. We will include experimental studies (randomized or cluster randomized and nonrandomized controlled trials, including quasi-randomized, controlled before-after, and interruptive time series) and observational studies, that is, including prospective cohort and case-control studies. The experimental and observational studies will only be included in the presence of control or comparison arms. Studies with a historical control arm will be excluded. The systematic review software, Covidence (Ventas Health Innovation), will be used to screen and select the studies. Further, 2 independent reviewers will complete the first and second levels of screening of studies and any conflicts arising will be resolved by consensus between the 2 reviewers or by involving the third reviewer. We will conduct the quality assessment of all included studies using the Risk of Bias tool for randomized controlled trials and nonrandomized controlled trials, and AMSTAR2 for systematic reviews.

**Results:**

Papers screening, data extraction, and synthesis will be completed by March 2024. We will use EPPI-Mapper (The International Public Policy Observatory) software to generate an online evidence map and to produce the tables and figures for the descriptive report. This EGM review will identify areas with high-quality, evidence-based digital KT tools (for immediate scale and spread) and areas where few or no KT tools exist (for targeted KT tool development and research or policy prioritization).

**Conclusions:**

This protocol focused on mapping eHealth KT tools that have been used in the literature to address SRH among adolescents. This will be the first EGM exercise to map digital KT tools to promote adolescents’ SRH and will incorporate a range of published sources.

**International Registered Report Identifier (IRRID):**

DERR1-10.2196/55081

## Introduction

### Background

Adolescence is a critical period in the transition from childhood into adulthood, during which young individuals aged 10 to 19 years experience substantial physical, psychological, social, and emotional changes [1]. Adolescents are vulnerable because of their age-related psychosocial and biological changes and the challenges associated with navigating these changes [2]. As part of their physical, psychological, and social development, it is common for adolescents to explore their sexual orientations and feelings through sexual experimentation [3]. Depending on their knowledge about sexuality and social support, some may experience unintended consequences such as unplanned pregnancy and sexually transmitted infections. Neglect of specific adolescents’ sexual and reproductive health (SRH) needs can affect their physical and mental health, future employment, economic well-being, and ability to reach their full potential [4-6].

SRH issues pose serious concerns for adolescents including the lesbian, gay, bisexual, transgender, queer, and gender diverse population. Adolescents have the highest rates of contracting sexually transmitted infections, including HIV [7,8]. Approximately 16 million girls aged 15-19 years and 2 million girls younger than 15 years become pregnant each year around the world [9]. Pregnancy-related complications are the second leading cause of death among girls aged 15-19 years [10]. Furthermore, adolescents have less access to information and services. Access to contraception also remains low among females 15-24 years compared to women older than 30 years [11]. This is primarily due to a lack of awareness, social stigma, policies, and procedures inhibiting the provision of contraception and abortion services to girls, and judgmental attitudes of health care professionals [12-14]. Young people have special SRH education needs that remain unmet, and to address these specific SRH needs, innovative and novel approaches are required to ensure access to evidence-based SRH information and acceptable SRH services [13].

To maximize health system resources and improve SRH outcomes for adolescents, it is increasingly important to close the research-practice gap by ensuring that research knowledge translates into action—a process called knowledge translation (KT). KT is the synthesis, exchange, and application of knowledge to improve the health of individuals, provide more effective health information, services, and products, and strengthen health care systems [15]. KT is the process of translating knowledge into action to provide more effective health services and strengthen the health care system [15,16]. The goal of KT is to ensure that individuals, such as patients, health professionals, and policymakers, can access and use research evidence to inform their health-related decision-making [17].

KT is a dynamic process that uses tools and methods to increase the use of evidence in decision-making while considering policy rationales in the process of generating evidence [18]. On the other hand, KT tools are a subgroup of KT interventions that facilitate moving research-based information to clinicians and patients or health care consumers in user-friendly language and formats to provide explicit recommendations or meet knowledge or information needs [16,17,19]. KT tools to transfer health information to health care consumers can be presented using a multitude of formats, including but not limited to videos, infographics, apps, practice guidelines, and decision-aiding tools to aid consumers in the synthesis, dissemination, and implementation of research [19]. Digital KT tools offer a promising approach to improving health literacy and communicating complex health information to health care consumers. Digital KT tools such as mobile phone websites, mobile apps, mobile health (mHealth), SMS text messaging, and social media platforms have been identified as useful public health tools, particularly to promote SRH among adolescents. The use of digital technology for SRH promotion offers privacy [20-25], access to personalized information [20,22,24,26], and convenience [20,25,27] making it a valuable way to provide accurate information about sexual health to adolescents [20-24].

Several research studies tested the effect of technology-based tools on improving adolescents’ knowledge, attitudes, and practices regarding sexual health [22-27]. Cornelius and Lawrence [23] used text messaging through mobile phone technology to increase awareness of adolescents to prevent sexually transmitted infections (HIV) and improve safe sexual practices [28]. The study reported that adolescents found text messaging to be an excellent source of information exchange and a reminder to have safe sex practices [27]. Another study evaluated the prevalence and effectiveness of using chatbot (chat agent) inquiries related to sex, drugs, and alcohol use among adolescents. The study reported that adolescents found chatbots a confidential and user-friendly tool to answer their queries related to topics around sex and sexual activities [28]. Additionally, Guse and colleagues [21] conducted a systematic review to summarize evidence on the effectiveness of digital media-based sexual health intervention for adolescents aged 13-24 years [29]. The review included 10 studies in total, while the majority of the included studies were conducted in the United States. The findings of the review study reported the effectiveness of digital media-based interventions in improving sexual behavior and health among adolescents.

However, despite the availability of digital KT tools, there is a lack of evidence of synthesis on the available digital or mHealth KT tools to promote SRH interventions for adolescents. Not all KT tools are equally effective and trustworthy sources of SRH information. Therefore, this study using an evidence gap map (EGM) exercise aims to synthesize evidence on the usefulness of digital KT tools to improve adolescents’ SRH. EGM is an emerging approach to mapping and synthesizing evidence from empirical studies to implement effective strategies and inform policymakers [30]. EGM is a systematic way of gathering scientific evidence and summarizing the evidence in a graphical format for researchers and practitioners to understand what is known and where the gap is in a particular sector. Another key advantage of using the EGM framework is that this guide is designed for nonspecialist audiences to distill key findings and provide implications for practice and policy [30].

### Study Purpose and Objective

This review aims to identify, map, and describe existing empirical evidence on the digital KT tools developed to improve adolescent SRH outcomes globally. In this EGM, we will assess and report on empirical studies describing the development, implementation, or evaluation of SRH digital KT tools to identify current aims, processes, methods, frameworks, tools, user roles, disciplinary and geographic domains of application, as well as to document existing knowledge gaps. The specific objectives of this EGM are to, first, identify, assess, and report on empirical studies of any design that describe the development, implementation, or evaluation of adolescent SRH digital KT tools; second, to identify current uses, purposes, and methods in the development of digital KT tools; third, to describe the characteristics of digital KT tools; and finally, to identify research gaps in the literature.

We will conduct an EGM to address the objectives, including reviewing relevant literature and a landscape analysis of the outcomes of interest. The EGM will be presented on a web-based platform and accompanied by a published paper with an analysis of the available evidence. An EGM aims to establish what we know and do not know about the effects of interventions in a thematic area [31]. The map is populated through systematic searching, screening, and data extraction of all relevant completed, and ongoing, impact evaluations and systematic reviews.

## Methods

### Study Design

This study is conducted in alignment with standard methodologies for the development of EGM [30]. The protocol for this work is registered with PROSPERO (CRD42022373970). We started this review in May 2023 and will complete it by April 2024.

### Scope of EGM

The scope of the EGM was determined through a consultative workshop organized by SM to discuss the interventions and strategies to improve the adolescents’ SRH and rights and on how to meet the information needs of adolescents related to SRH. We invited the stakeholders from Option for Sexual Health and Action Canada for Sexual Health and Rights to the consultative workshops for research team members of World Universities Network and members of the Contraception and Abortion Research Team. This workshop identified the need for the EGM and informed the general inclusion criteria and the framework of the EGM. We found EGM as the most appropriate and suitable framework for this review as we aim to map the available evidence on the effectiveness of digital interventions in the form of a table or matrix that provides a visual presentation of the evidence [32].

### Eligibility Criteria

The eligibility criteria for the evidence map are summarized in Textbox 1 and detailed below:

- *Study types:* We will include experimental studies (randomized or cluster randomized and nonrandomized controlled trials, including quasi-randomized, controlled before-after, and interruptive time series) and observational studies, that is, including prospective cohort and case-control studies. The experimental and observational studies will only be included in the presence of control or comparison arms. Studies with a historical control arm will be excluded. Studies such as cross-sectional studies, case reports and series, editorials, commentaries, and narrative reviews will not be included.
- *Topics of interest:* We will include studies that have reported KT tools on SRH topics such as family planning and contraception use, healthy timings and spacing of pregnancy, teenage pregnancy, abortion, HIV and AIDS and other sexually transmitted infections, intimate partner violence or dating violence, menstruation, feminine hygiene, child marriage, and female genital mutilation associated with the above topics.
- *Population:* We will include primary studies conducted on adolescents aged 10-19 years. Studies and reviews on populations younger and older than adolescents but inclusive of adolescents will be included if they separately report outcomes on adolescents. We will report the studies separately if they do not report outcomes on adolescents separately.
- *Exposure or intervention:* We will include studies assessing digital KT tools for disseminating SRH information to adolescents. If a digital KT tool is meant for dissemination in wider age groups, it will be included, and these KT tools will consist of websites, mobile apps, mHealth, SMS text messaging, pamphlets, and brochures over email, podcasts, mass media such as radio messages or videos on television, and social media such as Instagram, TikTok, Facebook, Twitter, YouTube, and over-the-top platforms.
- *Comparison:* We will include studies comparing the abovementioned KT tools with no interventions, the standard of care, or other interventions.
- *Setting:* We will include studies conducted globally regardless of the settings or context of its conduct.

**Eligibility criteria for the evidence map.**

**Population**

- Adolescents aged 10-19 years

**Exposure or intervention**

- Digital knowledge translation tools for disseminating sexual and reproductive health information such as websites, mobile apps, mobile health (mHealth), SMS text messaging, pamphlets, and brochures over email, podcasts, radio messages, videos on television, and social media such as Instagram, TikTok, Facebook, and Twitter

**Comparison**

- Any comparison is eligible

**Geography**

- Global evidence, that is, studies from high-income and low- and middle-income countries

**Topics of interest**

- Family planning and contraception use
- Healthy timings and spacing of pregnancy
- Teenage pregnancy
- Abortion
- HIV and AIDS and other sexually transmitted infections
- Intimate partner violence or dating violence
- Menstruation and feminine hygiene
- Child marriage
- Female genital mutilation
- Rights, norms, education, and empowerment associated with the above topics

**Study type**

- Experimental and observational studies, including systematic reviews

**Timeframe**

- Studies and reports published from 2010 onwards as internet and digital or social media usage increased from 2010

**Language**

- English language only given the prominence of the English language in science

### Search Strategy

The search strategy for this review will be reported in adherence to the PRISMA-S (Preferred Reporting Items for Systematic Reviews and Meta-Analyses for Searching) extension [33]. The search strategy will be developed by an experienced health sciences librarian (MK) in consultation with the research team. The following databases will be searched individually from inception to present: MEDLINE (1946-present), Embase (1974-present), and Global Health (1910-present) via OVID; CINAHL (1936-present) via EBSCOhost; Scopus (1976-present); and Cochrane Library (1993-present) via Wiley.

The search strategy will be derived from four main concepts: (1) adolescents, teenagers, or young adults; (2) SRH or health services including vocabulary related to contraception, family planning, pregnancy, sexually transmitted infections, and gender-based violence; (3) digital communication tools such as websites, web-based messaging, smartphones, mobile apps, social media, podcasts, television, or digital information; and (4) KT including vocabularies such as information dissemination, research innovation, knowledge transfer, implementation science, research into practice, knowledge into practice, and evidence-based practice. Bibliographic databases will be searched using a combination of natural language (keywords) and subject headings, such as Medical Subject Headings, wherever they are available. Items such as books, book chapters, editorials, conference materials, and opinion pieces will be removed from the results and a publication date limit of 2010 to the present will be applied. A preliminary search for OVID Medline was developed and executed in October 2022 to determine the feasibility of this project and test the scope (see Multimedia Appendix 1 for the MEDLINE search strategy). The systematic review software, Covidence, will be used to manage this review. Covidence will be used for the deduplication of database search results and will be used to facilitate the title or abstract screening and full-text screening phases.

### Screening and Extraction

All the studies identified from the databases will be imported to Covidence (a digital screening software), and 2 independent reviewers will screen the studies in title or abstract and full-text stages. Disagreements, if any, will be resolved by consensus. The reference list of all the included studies will be scanned and searched to include any relevant study that may have been missed during the search of databases. The final list of included studies will be subject to forwarding and backward citation chaining to ensure no relevant eligible papers have been missed. A diagram showing the flow of literature will be produced and reported by PRISMA (Preferred Reporting Items for Systematic Reviews and Meta-Analyses) guidelines [33]. For data extraction, we will use a standardized data extraction form to extract descriptive data from all studies meeting our inclusion criteria. Data extracted from each study will include bibliographic details, KT tool types and descriptions, outcome types and descriptions, study design, context or geographical information, and details on the outcome and quality of the KT tool. Further, 2 review authors will independently extract data, and discrepancies will be resolved through discussion until consensus has been achieved or by consulting a third reviewer if required. Attempts will be made to contact the authors of the included studies to obtain clarifications or additional data. We will extract data on the following study characteristics presented in Textbox 2:

**Extraction of data on this study’s characteristics.**

**Publication information**

- Journal, publication year, study location, and study setting

**Population**

- Number of participants, sex, mean age, age range, and sociodemographic status

**Digital knowledge translation (KT) tool**

- Digital KT tool description, purpose, setting, and any other important detail

**Intervention (if studies used digital KT tool as an intervention to improve sexual and reproductive health [SRH] information and outcome of adolescents)**

- Intervention description, the composition of the intervention, duration of intervention, a platform for delivery (school-based or community-based, peer-support, mobile health [mHealth]), and comparison group description

**Control**

- Standard of care or control

**Outcomes**

- Outcomes specified earlier, time points of outcomes reported, and evaluation methods used

**Study design**

- Type of study design

**Other information**

- Funding sources, study limitations, and conflicts of interest

### Data Synthesis

Data from the review will be visually synthesized in an EGM using EPPI-Mapper [34] with an accompanying narrative. Rows of the EGM list digital KT strategies and columns components of outcomes and other relevant data coding. Each cell shows the number and quality of evidence for digital KT strategies. We will conduct the quality assessment of the included studies using Risk of Bias for interventional studies including randomized controlled trials and nonrandomized controlled trials and AMSTAR2 tool for systematic reviews [31,35]. The EGM identifies areas with high-quality, evidence-based digital KT tools (for immediate scale and spread) and areas where few or no KT tools exist (for targeted KT tool development and research or policy prioritization) An example of the layout of the evidence map matrix can be found in Multimedia Appendix 2 (developed in consultation and discussion with experts in the area of adolescents SRH).

### Patient and Public Involvement

Patients will not be involved in the design, conduct, reporting, or dissemination of the EGM exercise.

### Ethical Considerations

No ethical review was required, as we included published data which is accessible and available for the public in this review. We will disseminate findings from the EGM through summary reports and publications; present our findings at conferences to facilitate research reach; list project descriptions and associated outputs on investigator-affiliated websites and professional web-based accounts; and concurrent dissemination through professional social media accounts. The EGM has the potential to inform researchers and decision makers about user-centered innovation by highlighting current practices and opportunities for further KT tools development. Findings will inform the development of future projects with the principal investigators and coprincipal investigators’ research program aimed at developing, implementing, and evaluating digital KT tools and associated projects in various receptive adolescent SRH contexts. Findings will directly inform a concurrent project by the lead investigator (SM) focused on co-designing a mobile app to improve the SRH of immigrant adolescents in Canada.

## Results

### Overview

Paper screening, data extraction, and synthesis will be completed by March 2024. We will use EPPI-Mapper software to generate a digital evidence map and to produce the tables and figures for the descriptive report. EPPI-Mapper will consist of rows representing digital KT interventions and column components presenting outcomes. Each cell shown within the matrix will present the number and quality of evidence for digital KT strategies. This EGM review will identify areas with high-quality, evidence-based digital KT tools (for immediate scale and spread) and areas where few or no KT tools exist (for targeted KT tool development and research or policy prioritization). This EGM exercise will offer a novel knowledge synthesis tool (a web-based EGM), mapping all of the digital KT tools currently available to promote the SRH of adolescents. The information will be available in a usable format to enable efficient and effective use by multi-stakeholder audiences.

## Discussion

### Principal Findings

The purpose of this review is to create an EGM to guide multi-sectoral stakeholders regarding digital KT tools to better understand and use the evidence-based digital KT tools to improve the SRH of adolescents. Digital KT tools have the potential to advance the SRH of adolescents, yet there is a dearth of knowledge regarding the quality and uptake of these digital KT tools. Digital technologies have the potential to facilitate innovative, efficient ways of meeting growing global health needs. A global 2019 report [36] indicated that over 5 billion mobile phone users, 4 billion internet users, and 3 billion active social media users, with most accessing the internet through mobile devices. The anonymity, convenience, and accessibility afforded by SMS, web access, and video streaming enhance the potential for SRH interventions to reach persons disconnected from mainstream SRH services.

Existing literature reviews on digital health interventions for adolescents’ SRH demonstrate limitations, including shortcomings in reporting and comprehensiveness that limit the utility and trustworthiness of findings. An EGM of digital KT tools is therefore warranted. Innovative ways to deliver sexual health information and services are more important than ever before due to the impact of the COVID-19 pandemic on access to SRH services globally [37]. Interruptions in SRH information and service delivery seriously impact vulnerable adolescents’ health and well-being. The absence of adolescent SRH services from “essential” health services during COVID-19 amplifies the potential role of technology in meeting the SRH needs of vulnerable groups like adolescents. Amidst the pandemic, technology has primarily provided the means to support the recalibration of health systems, service provision, and information delivery [38]. Digital health tools play an important role in preserving the continuity of SRH services for adolescents and youth during a crisis. Using digital health tools in the pandemic and climate change context would improve adolescents’ access to information which might support them in having the knowledge and motivation to access services.

Guidelines and policies are increasingly focused on adolescents as a specific population for modifying health behaviors [39], and the global community is calling for further research in this area [40]. However, it is important to first map out all the available digital KT tools for improving behavior related to SRH among adolescents. Digital or eHealth KT interventions have been identified as useful public health tools, particularly to advance SRH among adolescents. The use of digital technology for SRH promotion offers privacy [21-26], access to personalized information [21,23,25,27], and convenience [21,26,28] making it a valuable way to provide accurate information about SRH to adolescents [21-26].

Despite the high level of SRH information consumption via a digital platform, evidence of the successful use of SRH digital KT tools is limited. EGM provides a novel knowledge synthesis approach that can accelerate the uptake of digital KT tools to promote adolescent SRH outcomes. This study will map all the digital KT tools currently available and identify the key evidence gaps in digital KT tools that may not be obvious from quick snapshots of the literature. Systematically mapping evidence for digital KT tools will provide valuable insights to inform practice, policy, research, or investment for future adolescent SRH research agendas.

### Strengths and Limitations of This Study

This will be the first EGM exercise to map digital KT tools to promote adolescents’ SRH and will incorporate a range of published literature sources. An independent dual-reviewer process will be used throughout to reduce the risk of bias and increase the likelihood of comprehensive study identification and inclusion. The 2D matrix of interventions and outcomes will help identify and inform policy and funding agencies for further specific areas of research. Publication bias is a possible limitation as non-English and gray literature will be excluded, which may underrepresent the digital KT tools available and used across geographically diverse contexts.

### Conclusions

This project will offer a novel knowledge synthesis tool (a digital EGM), mapping all of the digital KT tools currently available to promote the SRH of adolescents. The information will be available in a usable format to enable efficient and effective use by multi-stakeholder audiences. This work is timely as innovative ways to deliver SRH information are more important than ever before due to the impact of digital media on accessing SRH information and services globally. In addition, key policies will be enacted to encourage and support research initiatives in underrepresented regions as this would promote a more comprehensive global understanding of SRH among adolescents. This review will report on the gaps identified in the research and quality assessment of included studies and recommendations will be set forth for future robust research studies. We will disseminate the findings of our study, via community symposiums, and public training sessions, and present at academic conferences and publish in open-access, peer-reviewed journals, advancing the body of knowledge on available digital KT tools to promote SRH and the rights of adolescents.

## Appendix

Multimedia Appendix 1 Preliminary Medline search strategy.

Multimedia Appendix 2 Example evidence map matrix.

## Abbreviations

EGM: evidence gap map
KT: knowledge translation
mHealth: mobile health
PRISMA-S: Preferred Reporting Items for Systematic Reviews and Meta-Analyses for Searching
PRISMA: Preferred Reporting Items for Systematic Reviews and Meta-Analyses
SRH: sexual and reproductive health
