# Supplementary material for: Digital Knowledge Translation Tools for Disseminating Sexual and Reproductive Health Information to Adolescents: Protocol for an Evidence Gap Map Review
Source: JMIR Res Protoc. 2024 Feb 13;13:e55081. doi: 10.2196/55081 (PMC10900081; doi:10.2196/55081)
Supplement: Multimedia Appendix 1 [file resprot_v13i1e55081_app1.pdf]

Supplementary File 1 - Preliminary Medline Search Strategy

Ovid MEDLINE(R) ALL <1946 to May 11, 2022>
Date of search: April 12, 2023

1 child/ or adolescent/ or young adult/ or (Adolescen* or teen* or youth? or "young people" or "younger people" or "young adult?" or "young women" or "young men" or "school age*" or student? or "middle schooler?" or "high schooler?" or "secondary school" or p?ediatric*).mp. 4048999
2 Sexual Health/ or exp Sexual Behavior/ 118798
3 Reproductive Rights/ 1077
4 ("Sexual health" or "sexual behavio?r*" or "sexual health right?").mp. 104713
5 Reproductive Health/ 4568
6 exp contraceptive agents/ or exp contraceptive agents, female/ or exp contraceptives, oral/ or exp contraceptives, postcoital/ or exp contraceptive agents, hormonal/ or exp contraceptive agents, male/ 77369
7 contraceptive devices/ or exp contraceptive devices, female/ or exp intrauterine devices/ or exp intrauterine devices, medicated/ or exp contraceptive devices, male/ 26599
8 pregnancy/ or pregnancy in adolescence/ or pregnancy, unplanned/ or pregnancy, unwanted/ 949650
9 exp Abortion, Induced/ 41756
10 Fertility/ 43014
11 ("Reproductive health*" or "sexual health*" or contracepti* or condom? or "birth control" or IUD or "intrauterine device?" or "family planning" or pregnanc* or pregnant or abortion? or abstinen* or fertility or childbearing).mp. 1291278
12 Sexually transmitted diseases/ or exp Sexually transmitted diseases, bacterial/ or exp Hepatitis C/ or exp HIV/ or (("sexual* transmi*" adj4 infection*) or "sexual* transmi* disease*" or STI or STIs or STD or STDs or STBBI or STBBIs or HIV or AIDS or hepatitis or HepC or chlamydia or gonorrh?ea or syphili*).ti,ab,kw. 740820
13 Sex Education/ 9127
14 sex* education.mp. 14886
15 gender-based violence/ or intimate partner violence/ or physical abuse/ or rape/ or Sex Offenses/ 21710
16 ("gender-based violence" or "dating violence" or "partner violence" or "gender-based abuse" or "dating abuse" or "partner violence" or "consensual sex" or rape* or "safe sex").mp. 35309
17 health services accessibility/ or right to health/ 83346
18 exp Reproductive Health Services/ 42767
19 ("sexual health clinic?" or "sexual health services" or "sexual health screen*" or "sexual health test*" or "sexual health check-up?").mp. 1710
20 menstrual cycle/ or menstruation/ 29087
21 exp Feminine Hygiene Products/ 2024

22 (menstruat* or menstrual or "female hygiene" or "feminine hygiene" or tampon? or (sanitary adj1 (pad? or napkin* or towel*)) or douch* or "vagin* wash*" or "vagin* clean*").mp. 71916
23 Marriage/ and (child* or underage* or minor* or adolescen* or teen).ab. 5353
24 ((child* or underage* or minor* or adolescen* or teen) adj2 (marriage or marry or married or marries)).mp. 1831
25 Circumcision, Female/ 1583
26 ("female genital mutilation" or "female circumcis*").mp. 1678
27 Sexual Partners/ 19485
28 exp Coitus/ 7793
29 ("sex* partner*" or "sexual intercourse" or "sexual relationship*" or coitus).mp. 48352
30 Sex Workers/ 2763
31 ("sex work*" or "transactional sex*" or prostitut* or "sex* slave*").mp. 13311
32 exp perinatal care/ or preconception care/ or prenatal care/ 42250
33 (prenatal or pre-natal or prepartum or pre-partum or preconception or pre-conception or perinatal or antenatal or ante-natal or antepartum or ante-partum or postnatal or post-natal or postpartum or post-partum).mp. 443270
34 or/2-33 2372280
35 1 and 34 432850
36 ((digital or virtual or online or internet or web-based) adj4 (translation or shar* or communicat* or inform* or messag*)).mp. 24771
37 Communication/ and (digital* or virtual* or online or internet or web-based).ab. 5210
38 exp Internet/ 92304
39 mobile applications/ or web browser/ 11351
40 blogging/ or exp mass media/ or radio/ or exp cell phone/ or television/ 68437
41 Electronic Mail/ 2893
42 (website* or internet or virtual* or online or web-based or web or digital*).mp. 722254
43 ("mobile app*" or smartphone* or "smart phone*" or "cell phone*" or cellphone* or "mobile phone" or iPhone* or iPad or android or Samsung or apple or mHealth or "text messag*" or "SMS messag*" or iMessage*).mp. 70657
44 (email* or "electronic mail*" or "electronic messag*").mp. 14468
45 ((digital* or virtual* or online or internet or web* or electronic*) adj3 (brochure* or pamphlet* or booklet* or leaflet* or handout* or hand-out* or flyer* or information or literature)).mp. 26697
46 (podcast* or webcast* or "web cast*" or radio or video? or television or TV or YouTube or Spotify or Tidal or "amazon music" or "amazon video" or iTunes or "google music" or "google video" or "google store").mp. 299979
47 ("social media" or instagram or Facebook or TikTok or "tik Tok" or twitter or Tumblr or WhatsApp or Snapchat or reddit or Pinterest or LinkedIn or WeChat or QQ or QZone or "Baidu tieba" or Viber).mp. 31872
48 or/36-47 1091922

49 translational medical research/ or diffusion of innovation/ or quality improvement/ or models, organizational/ or health impact assessment/ or program planning/ or health services research/ 115861

50 ((translat* or implement* or adopt* or uptake or scale) and (change* or improv* or research or knowledge or information or innovat* or evidence or outcome*)).mp. 1896397

51 (((change or improv* or implement*) adj3 (manag* or strateg* or success*)) or (quality adj1 improv*)).mp. or (chang* or improv*).ti. 1076004

52 Organizational Innovation/ or exp Health Services Accessibility/og, fs 35979

53 (((Knowledge or evidence or research or innovation or information) adj4 (translat* or transfer* or mobiliz* or mobilis* or exchange or implement* or disseminat* or utiliz* or utilis* or uptake or adopt* or communicat* or sharing or educat*)) or "research into practice" or "evidence into practice" or "knowledge to action" or "knowledge gap*" or "research to policy" or "translational science" or "implementation science" or "decision making" or "policy making").mp. 519716

54 exp Evidence-Based Practice/ 92873

55 ("health* innovation*" or "health care innovation*").mp. 1074

56 ("evidence-based" or "evidence-informed" or EBP or EBM or EBI).ti,ab. 148096

57 or/49-56 3339862

58 35 and 48 and 57 5898

59 limit 58 to yr="2012 -Current" 4694

60 (editorial or letter or note or opinion).pt. 1784211

61 59 not 60 4668
