# Supplementary material for: Digital Knowledge Translation Tools for Disseminating Sexual and Reproductive Health Information to Adolescents: Protocol for an Evidence Gap Map Review
Source: JMIR Res Protoc. 2024 Feb 13;13:e55081. doi: 10.2196/55081 (PMC10900081; doi:10.2196/55081)
Supplement: Multimedia Appendix 2 [file resprot_v13i1e55081_app2.docx]

**Multimedia Appendix 2.** Example evidence map matrix.

| **Intervention Categories**  (digital KT strategies) | **Outcome Categories** | **Adolescent knowledge, attitudes, and empowerment** | | | | **Adolescent behaviours** | | | | **Adolescent health** | | | | | **Health services** |
| --- | --- | --- | --- | --- | --- | --- | --- | --- | --- | --- | --- | --- | --- | --- | --- |
|  |  | Knowledge and awareness | Attitudes | self-efficacy | normative change | Sexual behaviour | Contraception and other prevention | Menstrual hygiene | Communication and support seeking | Pregnancy and births | Abortion | HIV/AIDS testing and incidence | Sexual & intimate partner violence | Other health outcomes | Accessing and utilizing services |
| Websites |  |  |  |  |  |  |  |  |  |  |  |  |  |  |  |
| Mobile app |  |  |  |  |  |  |  |  |  |  |  |  |  |  |  |
| mHealth |  |  |  |  |  |  |  |  |  |  |  |  |  |  |  |
| SMS/text messages |  |  |  |  |  |  |  |  |  |  |  |  |  |  |  |
| Digital Pamphlet/  brochure |  |  |  |  |  |  |  |  |  |  |  |  |  |  |  |
| OTT media (Netflix, Prime, YouTube, etc. |  |  |  |  |  |  |  |  |  |  |  |  |  |  |  |
| Social Media (Facebook, Instagram, WhatsApp, TikTok, SnapChat, LinkedIn) |  |  |  |  |  |  |  |  |  |  |  |  |  |  |  |
| Other ICT |  |  |  |  |  |  |  |  |  |  |  |  |  |  |  |
| Radio/TV |  |  |  |  |  |  |  |  |  |  |  |  |  |  |  |
| Podcast |  |  |  |  |  |  |  |  |  |  |  |  |  |  |  |
